# Excessive whole-body exposure to 28 GHz quasi-millimeter wave induces thermoregulation accompanied by a change in skin blood flow proportion in rats

**DOI:** 10.3389/fpubh.2023.1225896

**Published:** 2023-09-04

**Authors:** Etsuko Ijima, Sachiko Kodera, Akimasa Hirata, Takashi Hikage, Akiko Matsumoto, Tatsuya Ishitake, Hiroshi Masuda

**Affiliations:** ^1^Department of Environmental Medicine, Kurume University School of Medicine, Kurume, Japan; ^2^Department Electrical and Mechanical Engineering, Nagoya Institute of Technology, Nagoya, Japan; ^3^Faculty of Information Science and Technology, Hokkaido University, Sapporo, Japan; ^4^Department of Social and Environmental Medicine, Saga University School of Medicine, Saga, Japan

**Keywords:** 5G, quasi millimeter-wave, whole body exposure, skin temperature, skin blood flow

## Abstract

**Introduction:**

Limited information is available on the biological effects of whole-body exposure to quasi-millimeter waves (qMMW). The aim of the present study was to determine the intensity of exposure to increase body temperature and investigate whether thermoregulation, including changes in skin blood flow, is induced in rats under whole-body exposure to qMMW.

**Methods:**

The backs of conscious rats were extensively exposed to 28 GHz qMMW at absorbed power densities of 0, 122, and 237 W/m^2^ for 40 minutes. Temperature changes in three regions (dorsal and tail skin, and rectum) and blood flow in the dorsal and tail skin were measured simultaneously using fiber-optic probes.

**Results:**

Intensity-dependent temperature increases were observed in the dorsal skin and the rectum. In addition, skin blood flow was altered in the tail but not in the dorsum, accompanied by an increase in rectal temperature and resulting in an increase in tail skin temperature.

**Discussion:**

These findings suggest that whole-body exposure to qMMW drives thermoregulation to transport and dissipate heat generated on the exposed body surface. Despite the large differences in size and physiology between humans and rats, our findings may be helpful for discussing the operational health-effect thresholds in the standardization of international exposure guidelines.

## 1. Introduction

In recent years, owing to remarkable progress in communication technology, wireless data communications such as Wi-Fi and smartphones have become ubiquitous and available worldwide. In addition, the future source of wireless communication is now moving to fifth-generation wireless communication systems (5G) to improve the quality of service by increasing data rates. 5G is planned to operate in frequency bands such as 24–28 GHz. However, the widespread use of 5G mobile networks has resulted in several problems. In particular, individuals are concerned about the potential adverse health effects of millimeter wave (MMW) exposure and some have suggested that the further deployment of 5G systems worldwide should be put on hold until more definitive studies on the safety of 5G systems are conducted, based on the precautionary principle (1).

The biological effects of electromagnetic field exposure have evaluated in experimental animals and humans. Kesari et al. (2) reported that 50 GHz-MMW exposure induced DNA double-strand breaks and chronically increased the apoptosis rate of cells during spermatogenesis (3) in rats. Other research groups have also showed that MMW exposure induces circulatory failure (4), hemorrhage and congestion of blood vessels (5), a decrease in the firing rate of the sural nerve (6), and gene expression of stress-induced transcription factors and heat-shock proteins (7). In contrast, Habauzit et al. (8) failed to find alterations in gene expression in rat skin after chronic exposure to 94 GHz-MMW. Of course, these studies were not performed under the same exposure conditions; these contradictory results should be resolved systematically. In addition, a recent review by Simko and Mattson reported limited results for 5G-MMW exposure, especially for exposure at 6–30 GHz (9).

The International Communication on Non-Ionizing Radiation Protection (ICNIRP) and IEEE International Commission on Electromagnetic Safety (ICES) have established guidelines to protect against substantiated adverse health effects. These guidelines have been revised in 2020 (10) and 2019 (11), respectively. Based on these international guidelines and standards, many countries have set exposure limits for both occupational and general public personnel. In these guidelines, the limits for whole-body averaged specific absorption rate (WBASAR) and absorbed (or epithelial) power density (APD) are set to protect against excessive core and local temperature increases at frequencies higher than 6 GHz, respectively. These metrics were set to relate to the core (12) and skin temperature rises (13). The revision extended the whole-body limit for whole-body exposure up to 300 GHz in the ICNIRP and 6 GHz in the IEEE ICES. In addition, the APD metric was introduced as basic restriction to prevent excessive skin heating instead of incident power density. The limit of the basic restriction of APD for local exposure in occupational and general public personnel are 100 and 20 W/m^2^, respectively, which was derived computationally in a conservative manner, corresponding to skin temperature of approximately 2.5°C and 0.5°C (14). These threshold temperatures were set such that exposure did not cause adverse health effects in the human body, considering the reduction (safety) factor.

Although these guidelines have been established, further experimental studies are required because the limits of the international guidelines were derived empirically without considering thermoregulation. In particular, it is important to experimentally determine the relationship between exposure intensity and skin temperature rise. In addition, the measurement of dynamic changes in skin blood flow is essential. MMWs penetrate only a few millimeters into the body surface (15, 16) and skin blood flow plays an important role in temperature regulation (17, 18). Therefore, if the thermoregulatory response—including the parameter changes under MMW exposure—can be elucidated, it would be useful to derive the limit in more scientific manner as well as the reduction factor.

The aim of this study was to determine the exposure intensity for increasing skin and rectal temperatures, and to investigate whether thermoregulation, especially for changes in skin blood flow, was induced during whole-body exposure to 28 GHz quasi-MMW (qMMW) in rats. Rats were chosen as experimental animals and simulated models to study the biological effects of electromagnetic field exposure. However, little information is available on their thermoregulation. We focused on the effects of exposure on thermoregulation in rats because they have highly sensitive thermoregulatory systems in their tails (19).

## 2. Materials and methods

### 2.1. Animals

Thirty-one male Sprague–Dawley rats (8–9 weeks old, Japan SLC, Japan) were used in this experiment. They were fed a standard pellet diet watered *ad libitum* in a room with a 12-h light/dark cycle at a temperature of 22.5 ± 1.0°C and a relative humidity of 50 ± 20%. The dorsal body hair of the rats were shorn 1–3 days before the start of the experiment. All experimental procedures were conducted in accordance with the ethical guidelines for animal experiments at Kurume University School of Medicine (approval numbers: 2020-174 and 2021-150).

### 2.2. Real-time measurements of physiological parameters

The temperature in three regions (rectum, dorsal skin, and tail skin) and the blood flow (BF) in two regions (dorsal skin and tail skin) were measured in real time using fiber-optic thermometers (FL-2400, Anritsu Meter Co., Ltd., Japan) and Doppler blood flow meters (FLO-C1 TWIN, Omegawave, Tokyo, Japan), respectively. The animals were anesthetized with isoflurane (3%) in 100% oxygen (Narcobit-E, Natsume Seisakusho Co., Ltd., Japan) for placement in an acrylic holder (Rev1, Kyoto Jushi-Seiko, Co., Ltd., Japan) and to set up the following probes: one thermometer probe was inserted into the rectum (2 cm from the anus), and the other two thermometer probes and BF-meter probes were placed in contact with the dorsal skin (below the antenna) and the base of the tail. All probes were made of non-metallic materials. Analog data acquired from the fiber-optic thermometers and Doppler BF meter were digitized using an A/D converter (PL3516, ADInstruments Ltd., New Zealand) at a sampling rate of 200 Hz (20). Because the Doppler BF meter was unable to measure the absolute flow values, the BF was revealed as a value relative to the reference response. This is displayed as the amount of change, with the value at the beginning of the exposure as 100%. BF data were analyzed after removing the upper 0.01 Hz with a low-pass filter.

### 2.3. MMW exposure equipment

**Figure 1:**
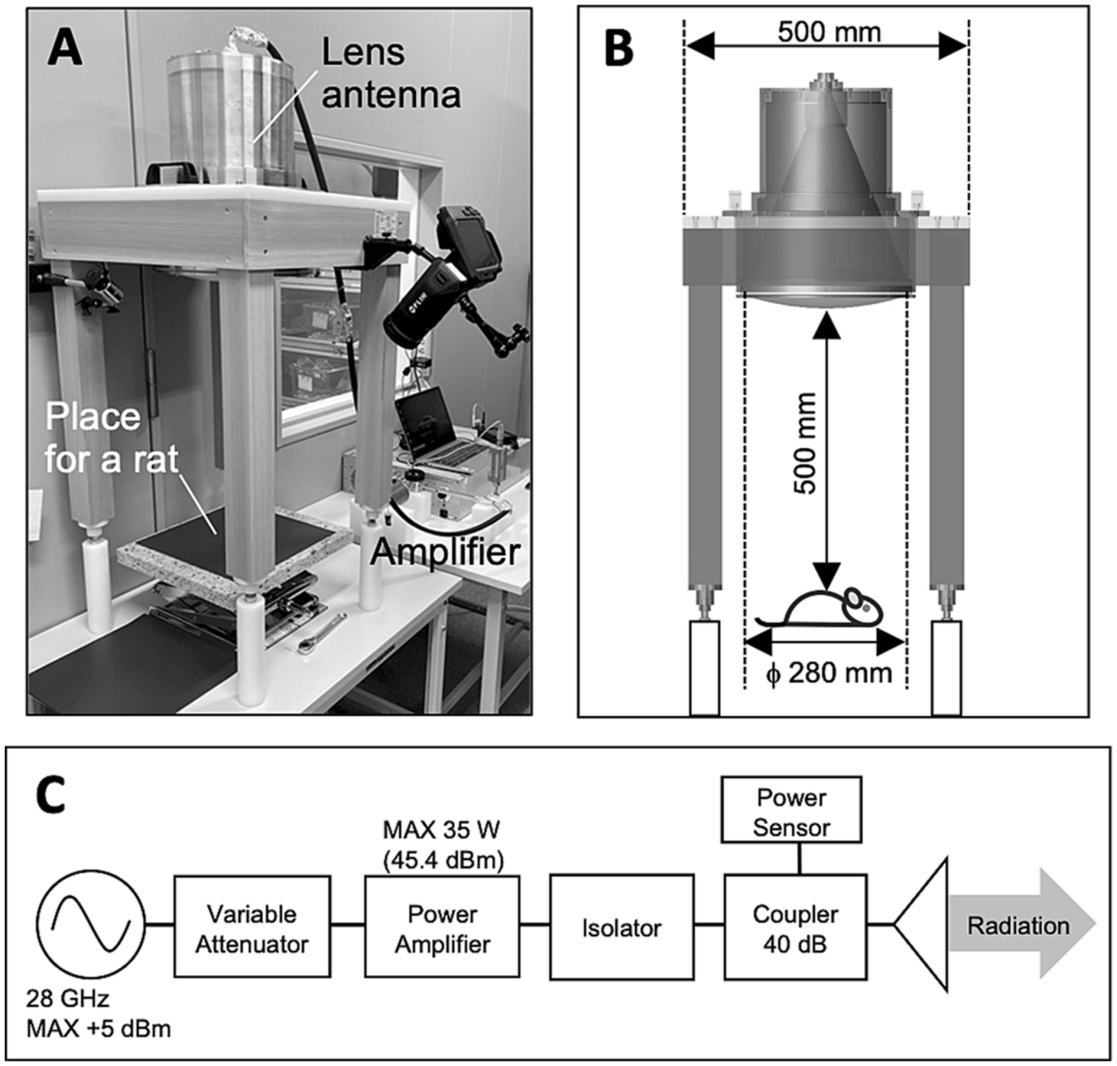
Exposure setup for animal experiment. **(A)** Overview of the exposure setup. **(B)** Only one rat was placed 50 cm below the center of the antenna surface for each experiment. **(C)** Block diagram of 28 GH-qMMW exposure equipment.

Whole-body exposure systems were designed to expose the rats to qMMW (Figure 1). The system consists of power supply chain as shown in Figure 1C: a signal generator (JOGSAG1401, SAF Tehnika, Riga, Latvia), an amplifier (AMP6034, Exodus Advanced Communications, Las Vegas, Nevada), a power meter (U8487A, Keysight Technologies, Inc., CA), and a lens antenna. The lens antenna consists of a conical-horn antenna and a dielectric lens which was made of the ultrahigh-molecular-weight polyethylene (relative permittivity of 2.3). The detailed of the developed antenna is described in our previous study (21). The sinusoidal wave at 28 GHz were generated using a signal generator and fed into an amplifier via a cable. The amplified continuous wave (CW) was fed to the lens antenna through a waveguide (WR-28) and power meter, and then radiated as a gaussian distribution on the surface 50 cm from the front of the antenna. The input power (IP) of the CW from the amplifier to the antenna was constantly monitored using a power meter via a 40 dB coupler. Three IP levels to the antenna, namely 0, 14, and 28 W, were selected for the experiment.

### 2.4. Computational method

**Figure 2:**
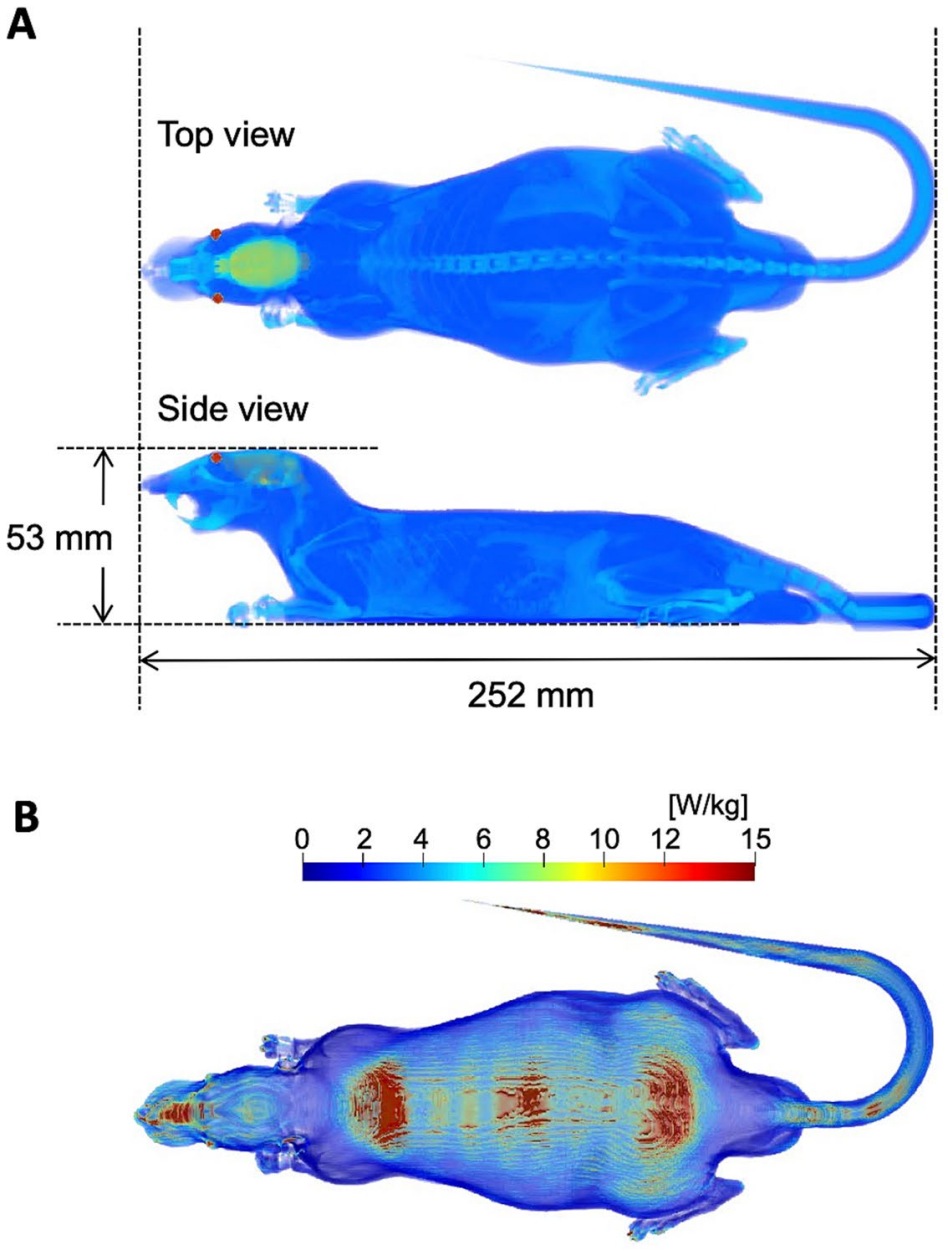
Dosimetric evaluation. **(A)** Numerical rat model. **(B)** SAR distribution on the rat model. The input power of antenna of the exposure system is 1 W.

Figure 2A illustrates a numerical rat model used to derive absorbed power in the computation (21, 22). The model was developed from CT image of a Sprague–Dawley rat weighing 265 g. The model consists of six anatomical tissues (skin, muscle, fat, bone, brain, and eye). The dielectric properties of the tissues were determined with a Cole–Cole dispersion model (23). The power absorption in the rat model was computed using the finite-difference time-domain (FDTD) methods (24) exposed from the lens antenna (21).

The WBASAR is defined as follows in the guideline (11):


(1) $$\mathrm{WBASAR}=\frac{\int \sigma \left(r\right){\left|E\left(r\right)\right|}^{2}dv}{\int \rho \left(r\right)dv}$$

where |***E***(***r***)|, *σ*, and *ρ*, ***r*** denotes the internal electric field (root mean square values), the conductivity, and the tissue density, position vector in the rat model, respectively.

The APD is defined at the body surface (11):


(2) $$\mathrm{APD}=\frac{1}{A}\iint_{A}ds\overset{z_{\max}}{\underset{0}{\int }}\sigma \left(r\right){\left|E\left(r\right)\right|}^{2}dz$$

where *A* denotes the averaging area, *z* = 0 is the body surface, *z*_max_ is the depth from the body surface, which is much larger than the penetration depth.

### 2.5. Experimental protocol

After setting for the measurements of the physiological parameters, a rat was kept in an acrylic holder without anesthesia and placed 50 cm below the antenna (Figure 1). 28 GHz-qMMW was exposed to the dorsal side of the rat for 40 min individually. Rats were randomly divided into three groups, and each group of rats was exposed at 0 W (*n* = 8), 14 W (*n* = 11), and 28 W (*n* = 12) of IP, respectively. The measurements started 4 min before the beginning of the exposure and lasted until the end of 40 min of exposure. The data, including the pre-exposure data, were recorded on a PC and later analyzed offline. Through the experiment, the room temperature and humidity were kept at 22.5 ± 0.4°C and 44.5 ± 1.3% (mean ± SD), respectively.

### 2.6. Statistical analysis

The difference in elapsed time per second compared to the sham-exposed group was analyzed nonparametrically using the Kruskal-Wallis test followed by the Steel test, a conservative method because the number of observations was insufficient to confirm the distribution. Estimation of exposure intensities of 28 GHz-qMMW from observations in rat exposure experiments at each exposure region was performed using a linear regression model based on the assumption of a normal distribution of temperature change (*p* = 0.1 to 0.9 for Shapiro–Wilk test). These tests were performed using Bell Curve for Excel software (ver. 3.21; Social Survey Research Information Co. Ltd.).

## 3. Results

### 3.1. Dosimetric evaluation in rat model

Figure 2 shows the distribution of power absorption in the rat model exposed to the lens antenna as an IP of 1.0 W. The peak spatial values of the incident power densities (IPD) and APD averaged over 4 cm^2^ corresponding to an IP of 1.0 W were 22.1 W/m^2^ and 8.5 W/m^2^, respectively, at 50 cm below the center of the antenna. Under the exposure conditions, the relative value of WBASAR was estimated as 0.26 W/kg. The IPD, APD, and WBASAR at each IP level are listed in Table 1.

**Table 1:** Exposure intensities.

Input power of antenna	IPD 50 cm below antenna center (W/m^2^)	APD at dorsal skin 50 cm below antenna center (W/m^2^)	WBASAR (W/kg)
1 W	22.1	8.5	0.26
14 W	318	122	3.7
28 W	616	237	7.2

The intensities of 28 GHz-qMMW exposure were set by the input power (IP) of the lens antenna. Corresponding values of incident power densities (IPD), absorbed power densities (APD), and whole-body averaged SAR (WBASAR) to the IP were calculated in computation using a numerical rat model.

### 3.2. Basal temperature conditions

At the beginning of whole-body exposure, the temperatures of the three regions were not significantly different among the three groups, including the sham-exposed group (*n* = 8–12 animals per group). The initial temperatures for all rats in dorsal skin, rectum, and tail skin were 34.9 ± 0.9°C, 36.7 ± 0.5°C, and 27.4 ± 1.5°C, respectively.

### 3.3. Trend over time of local temperature and blood flow changes

**Figure 3:**
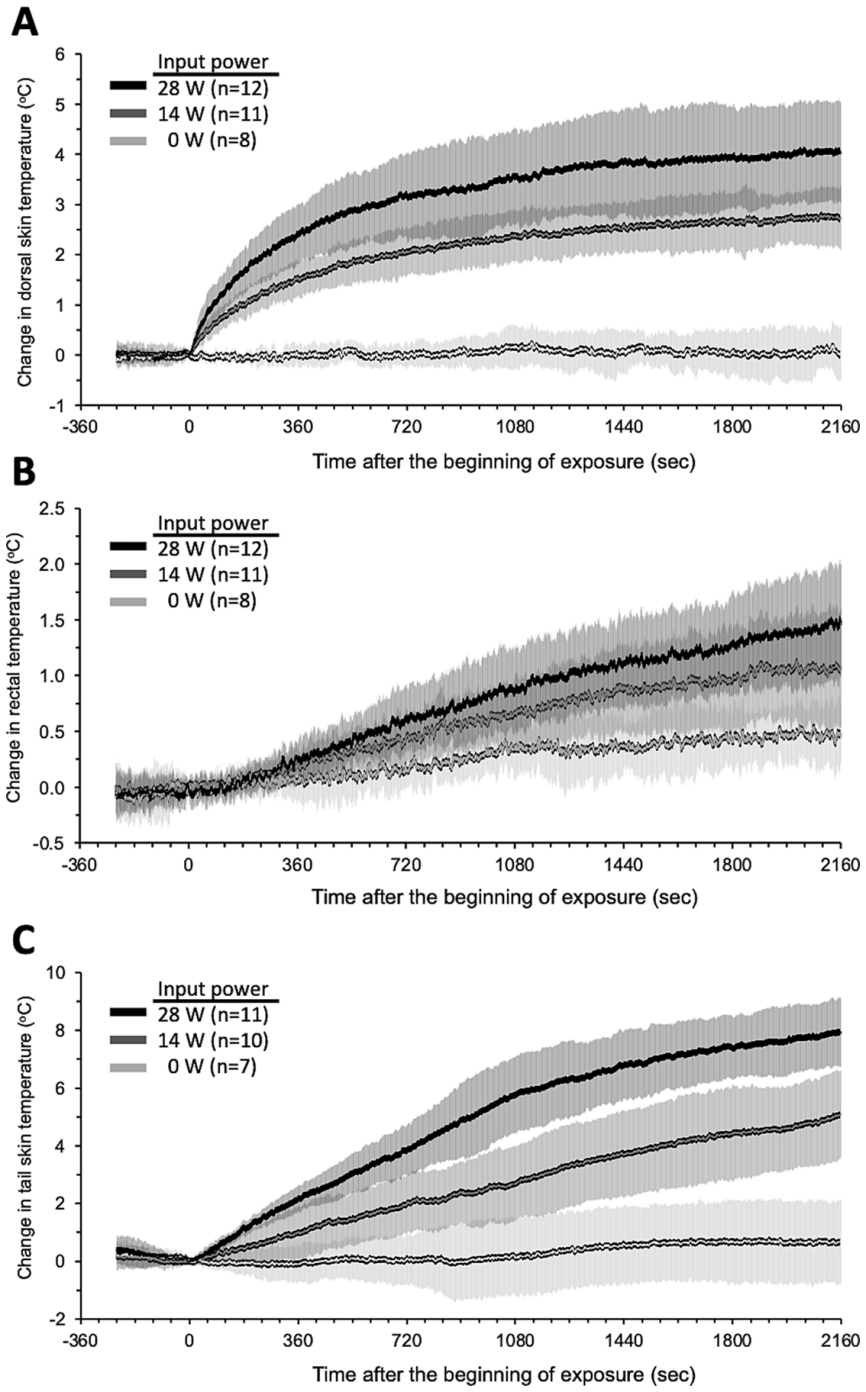
Trend over time of temperature changes during qMMW exposure. Temperature changes (°C) in the dorsal skin **(A)**, rectum **(B)**, and tail skin **(C)** were measured simultaneously throughout the experiments in conscious rats. The 28 GHz-qMMW exposure was started after 4 min of pre-recording. Graphs show mean ± SD.

Local temperature and BF were measured simultaneously during exposure to investigate whether qMMW exposure changes them. Figure 3 shows the trend over time of the temperature response to qMMW exposure. In the two qMMW-exposed groups, the dorsal skin temperature increased immediately after exposure (Figure 3A), whereas there was a delay of 46 s or more in the rectum (Figure 3B) and tail skin (Figure 3C). These differences were reflected in the elapsed time during which a significant increase in temperature was detected compared to the sham-exposed group (Table 2). The elapsed time for dorsal skin temperature was 4 s and 5 s for 28 W and 14 W IP, respectively. In contrast, 49 s and 351 s of elapsed time were required for the tail skin and rectum, respectively, even at 28 W of IP.

**Table 2:** Elapsed time to detect significance from sham-exposed group.

Region of body	Temperature change		Blood flow change	
	14 W	28 W	14 W	28 W
Dorsal skin	5	4	n.s	n.s
Rectum	355	351	–	–
Tail skin	190	49	847	570
				(sec)

Significant differences compared with sham-exposed group were statistically evaluated by Kruskal-Wallis test followed by Steel test every second. n.s., not significant.

These temperature increases persisted until the end of exposure, but the trend over time patterns were different. The dorsal skin temperature had two distinct phases. It approached its maximum value after the first phase of the rapid temperature rise, followed by the second phase of gradual temperature rise. In contrast, the rectal and tail skin temperatures increased almost linearly until the end of the exposure period. Interestingly, the temperature changes in the tail skin were approximately twice as large as those in the dorsal skin at the end of the exposure.

**Figure 4:**
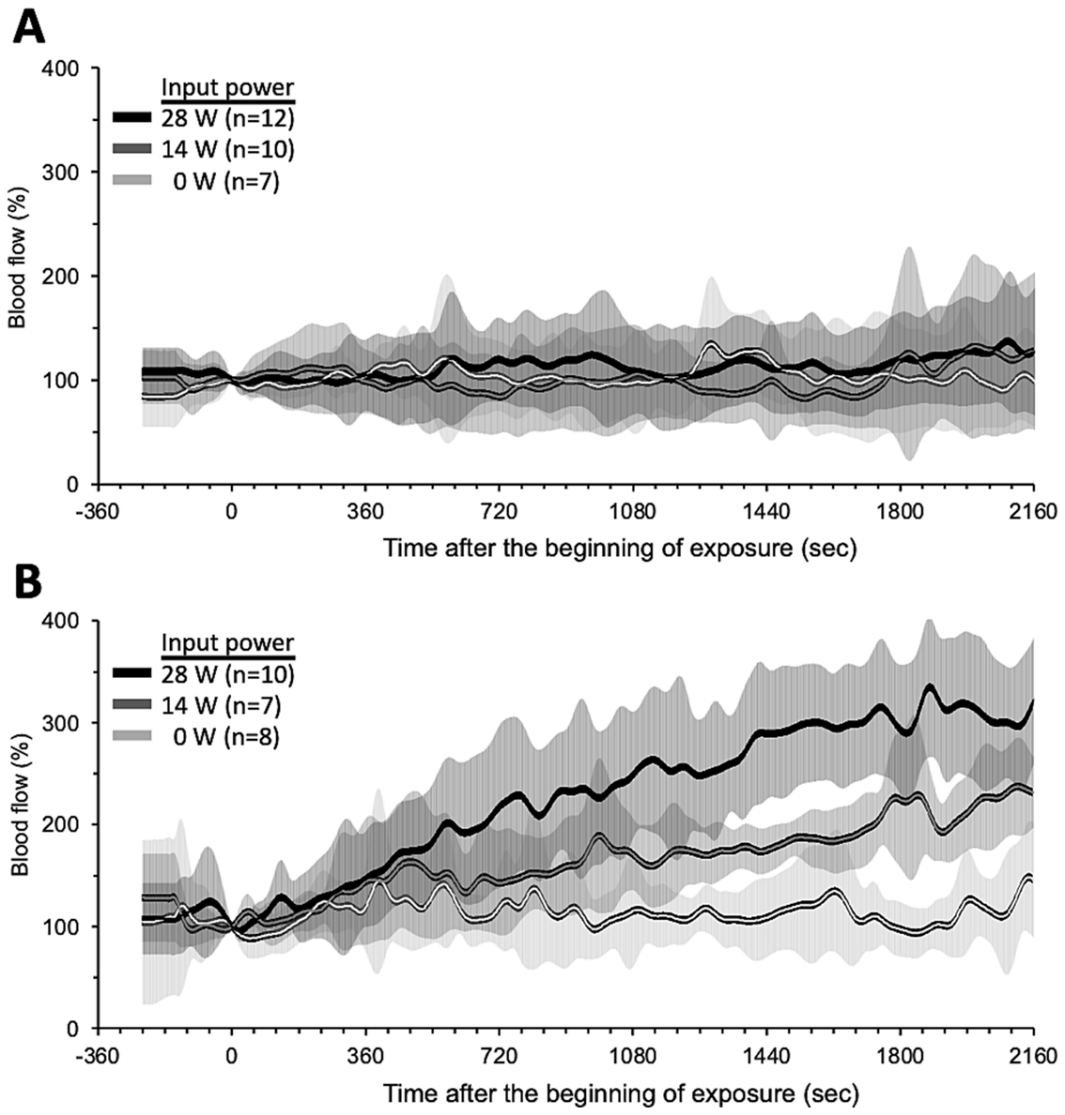
Trend over time of skin blood flow changes during qMMW exposure. Blood flow changes (%) in dorsal skin **(A)** and tail skin **(B)** were measured simultaneously throughout the experiments in conscious rats. The 28 GHz-qMMW exposure was started after 4 min of pre-recording. Graphs show mean ± SD.

Figure 4 shows the trend over time of the BF under qMMW exposure. Dorsal skin BF (Figure 4A) showed no obvious changes with exposure, even at the maximum intensity. In contrast, tail skin BF increased linearly from the beginning to the end of exposure (Figure 4B). In addition, the degree of increase and elapsed time of tail skin BF were dependent on the exposure intensity. Although changes in the BF were observed early after exposure, the elapsed time was more than 6 min because of the variability of BF signals, which are susceptible to rat movements.

### 3.4. Intensity-dependent changes in local temperature

**Figure 5:**
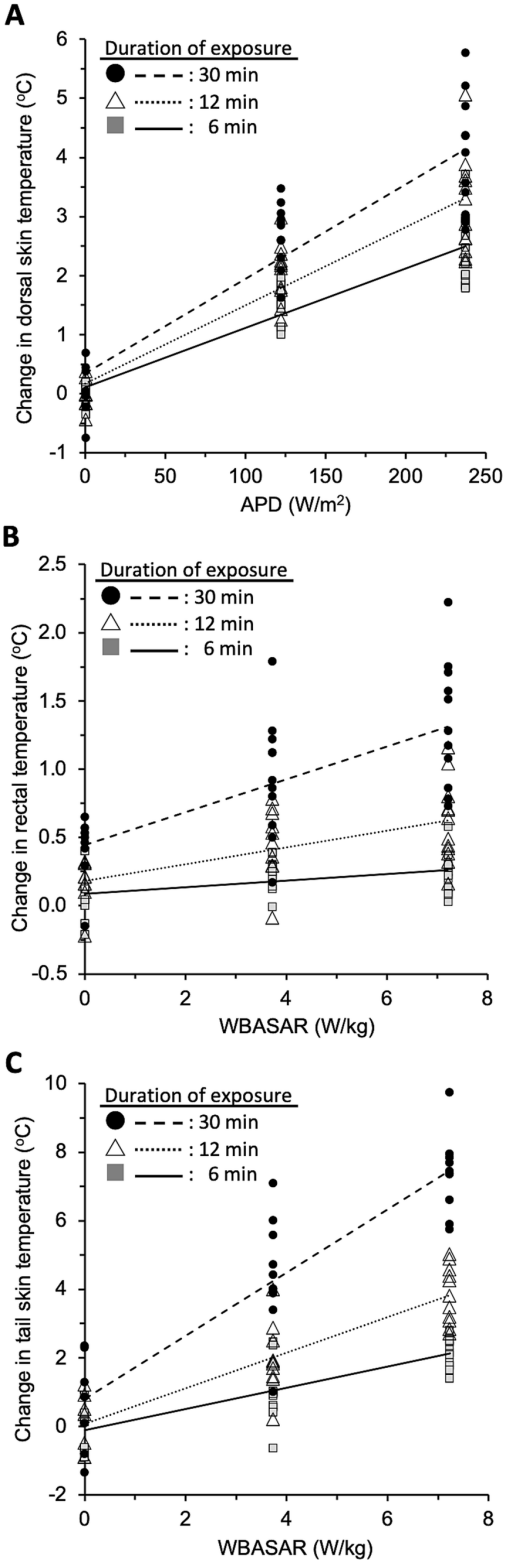
Intensity-dependent effect of qMMW exposure. The plots show the temperature values in three regions: dorsal skin **(A)**, rectum **(B)**, and FIGURE 5 (Continued)tail skin **(C)** at 6, 12, and 30 min after the beginning of 28 GHz-qMMW exposure. The exposure intensities were set as APDs of 0, 122, and 237 W/m^2^, and WBASARs of 0, 3.7, and 7.2 W/kg. Each line shows the linear regression between exposure intensity and temperature in dorsal skin (*n* = 31), rectum (*n* = 31), and tail skin (*n* = 28).

To elucidate whether the increase in local temperature depended on the intensity of qMMW exposure, we analyzed the correlation between these parameters at 6, 12, and 30 min after the beginning of exposure (Figure 5). At first, the normality of the data was confirmed for all combinations of exposure intensity and exposure duration in three regions. The temperature changes in all three regions had a significant relationship with the exposure intensity in each period, and the relationships were indicated as linear regression models (Table 3).

**Table 3:** Estimation of the exposure intensities of 28 GHz-qMMW from observations in rat experiments at each exposed region.

	Experimental observations			Regression models*	Estimations	
**Dorsal skin**						
	**Temperature change (°C)**				**APD (W/m^2^)**	
APD	0 W/m^2^	122 W/m^2^	237 W/m^2^	*n* = 31	for +1°C	for +5°C
6 min	0.0 ± 0.2	1.5 ± 0.3	2.4 ± 0.6	*y* = 0.010*x_a_* + 0.114, *R* = 0.91, *p* < 0.001	88	488
12 min	0.0 ± 0.3	2.1 ± 0.5	3.2 ± 0.8	*y* = 0.013*x_a_* + 0.176, *R* = 0.90, *p* < 0.001	62	365
30 min	0.1 ± 0.4	2.7 ± 0.5	3.9 ± 1.0	*y* = 0.016*x_a_* + 0.344, *R* = 0.89, *p* < 0.001	41	291
**Rectum**						
	**Temperature change (°C)**				**WBASAR (W/kg)**	
WBASAR	0 W/kg	3.7 W/kg	7.2 W/kg	*n* = 31	for +1°C	for +5°C
6 min	0.0 ± 0.2	0.2 ± 0.1	0.2 ± 0.1	*y* = 0.024*x_w_* + 0.085, *R* = 0.43, *p* = 0.016	37	201
12 min	0.2 ± 0.2	0.4 ± 0.3	0.6 ± 0.3	*y* = 0.061*x_w_* + 0.180, *R* = 0.59, *p* < 0.001	13	79
30 min	0.4 ± 0.2	0.9 ± 0.4	1.3 ± 0.5	*y* = 0.120*x_w_* + 0.443, *R* = 0.66, *p* < 0.001	4.6	38
**Tail skin**						
	**Temperature change (°C)**				**WBASAR (W/kg)**	
WBASAR	0 W/kg	3.7 W/kg	7.2 W/kg	*n* = 28	for +1°C	for +5°C
6 min	−0.1 ± 0.7	1.0 ± 0.9	2.2 ± 0.4	*y* = 0.311*x_w_* – 0.115, *R* = 0.80, *p* < 0.001	3.6	16
12 min	0.1 ± 0.9	2.0 ± 1.0	3.9 ± 0.8	*y* = 0.519*x_w_* + 0.087, *R* = 0.87, *p* < 0.001	1.8	9.5
30 min	0.7 ± 1.4	4.4 ± 1.6	7.4 ± 1.1	*y* = 0.920*x_w_* + 0.813, *R* = 0.89, *p* < 0.001	0.2	4.6

Number of observations = 7–12 for each group. APD, absorbed power densities; WBASAR, whole-body averaged SAR. *y = temperature change (°C), x_a_ = APD (W/m^2^), x_w_ = WBASAR (W/kg). The significant relationship between two factors was evaluated by Pearson’s correlation test, and a value of *p* < 0.05 was considered statistically significant. The exposure intensities to induce 1°C or 5°C of temperature increase in each region were estimated using the obtained regression models.

Using these regression models, we estimated the exposure intensities to induce 1°C or 5°C of temperature increase in each region. The dorsal skin was directly irradiated with qMMW; thus, the required exposure intensities were evaluated using a regression model between the APD value and temperature change in the dorsal skin. As a result, it was estimated that 88 W/m^2^ and 488 W/m^2^ of APD could induce 1°C and 5°C of temperature increase, respectively, for 6 min of exposure.

In contrast, rectal and tail skin temperatures were unlikely to be directly altered by qMMW exposure. Therefore, it is suitable to calculate the required intensity using the WBASAR value for 30 min exposure. It was estimated that 4.6 W/kg and 38 W/kg of WBASAR could induce 1°C and 5°C of rectal temperature increase, respectively. In tail skin, 0.2 W/kg and 4.6 W/kg of WBASAR were required for 1°C and 5°C temperature increase, respectively.

### 3.5. Correlation between local temperature and blood flow

Previous physiological studies in rats (19) suggest that heat accumulated in the rat body is dissipated through the tail. Indeed, in the present experiment, the largest temperature change was observed in the tail skin of the three regions we measured (Table 3), even though the tail region was not exposed to much qMMW. This fact reminds that a similar thermoregulation may be at work during dorsal skin heating by qMMW exposure. To explore the mechanism of this heat transfer, we plotted the mean ± SD values of temperature and blood flow changes in each region every 3 min after the beginning of exposure, and examined the correlation between the measured parameters.

**Figure 6:**
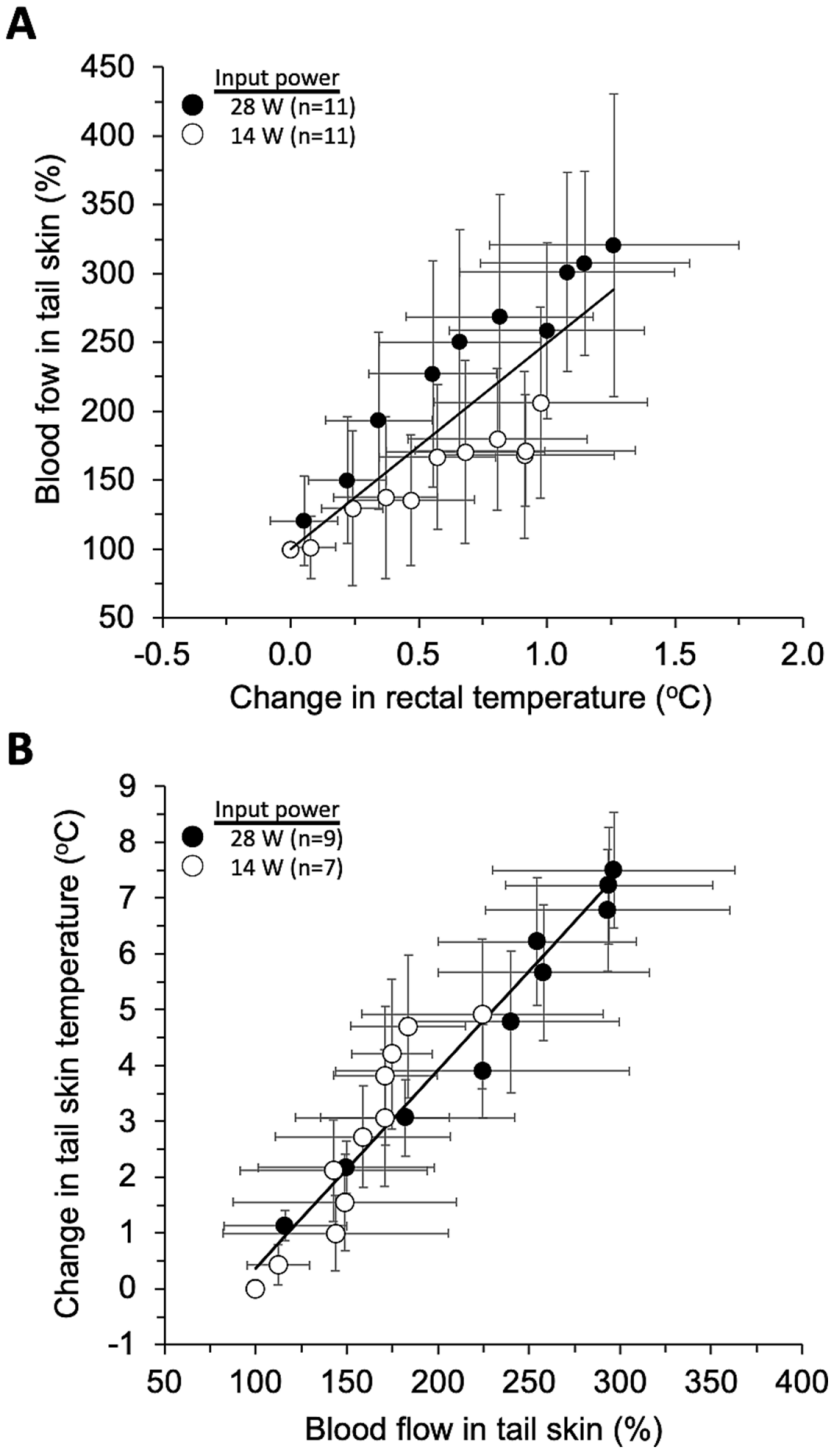
Models of thermoregulatory systems. The plots show the relationship between three parameters (mean ± SD) every 3 min after the start of exposure, and the line shows the linear regression model fitted using all plots including two exposure intensities. Rectal temperature changes-tail skin blood flow **(A)** and tail skin blood flow-tail skin temperature **(B)** were significantly correlated (*p* < 0.01).

As a result, two characteristic linear relationships with relatively little dependence on exposure intensity were found between rectal temperature rise and tail skin BF (Figure 6A), and between tail skin BF and tail skin temperature (Figure 6B). One was that the tail skin BF (BFt) increases in proportion to rectal temperature change (Tr), as shown by the equation BFt = 149.5Tr + 99.8 (*R*^2^ = 0.721, *p* < 0.01). This linear model was designated as thermoregulatory model-1 for later use. The other was that the tail skin temperature (Tt) rises in proportion to the change in tail skin BF (BFt), as shown by Tt = 0.036BFt – 3.18 (*R*^2^ = 0.933, *p* < 0.01). This second linear model was designated as the thermoregulatory model-2 for later use.

### 3.6. Estimation of blood flow and temperature changes in the tail skin

To validate the above two linear regression models in the rat thermoregulatory system, we estimated the increase in tail skin BF and the change in tail temperature based on the increase in body temperature during qMMW exposure and compared the estimated values with those obtained in animal experiments. Because a significant increase in tail BF was observed at least 570 s after the beginning of exposure (Table 2), estimates were made for data at 12 and 30 min of exposure.

First, rectal temperature changes during qMMW exposure were calculated for every 0.5 W/kg in the range of WBASAR from 0 to 8 W/kg, using a model derived from the relationship between WBASAR and rectal temperature change. In the animal experiment, the rectal temperature of the sham-exposed group was maintained below 0.5°C, but spontaneous temperature rises were observed throughout the experimental period. To avoid the influence of such spontaneous changes in the estimation using the regression model, the rectal temperature during the sham exposure was defined here as 0°C. In this case, the equations for rectal temperature change at 12 and 30 min were modified to *y* = 0.091x_w_ and *y* = 0.193x_w_, respectively, compared to the regression equations in Table 3. Where y and *x_w_* are rectal temperature change (°C) and WBASAR (W/kg), respectively. Using these equations, the rectal temperature change was estimated to be 0 to 0.7°C and 0 to 1.5°C at 12 and 30 min of exposure, respectively, for WBASAR 0 to 8 W/kg.

**Figure 7:**
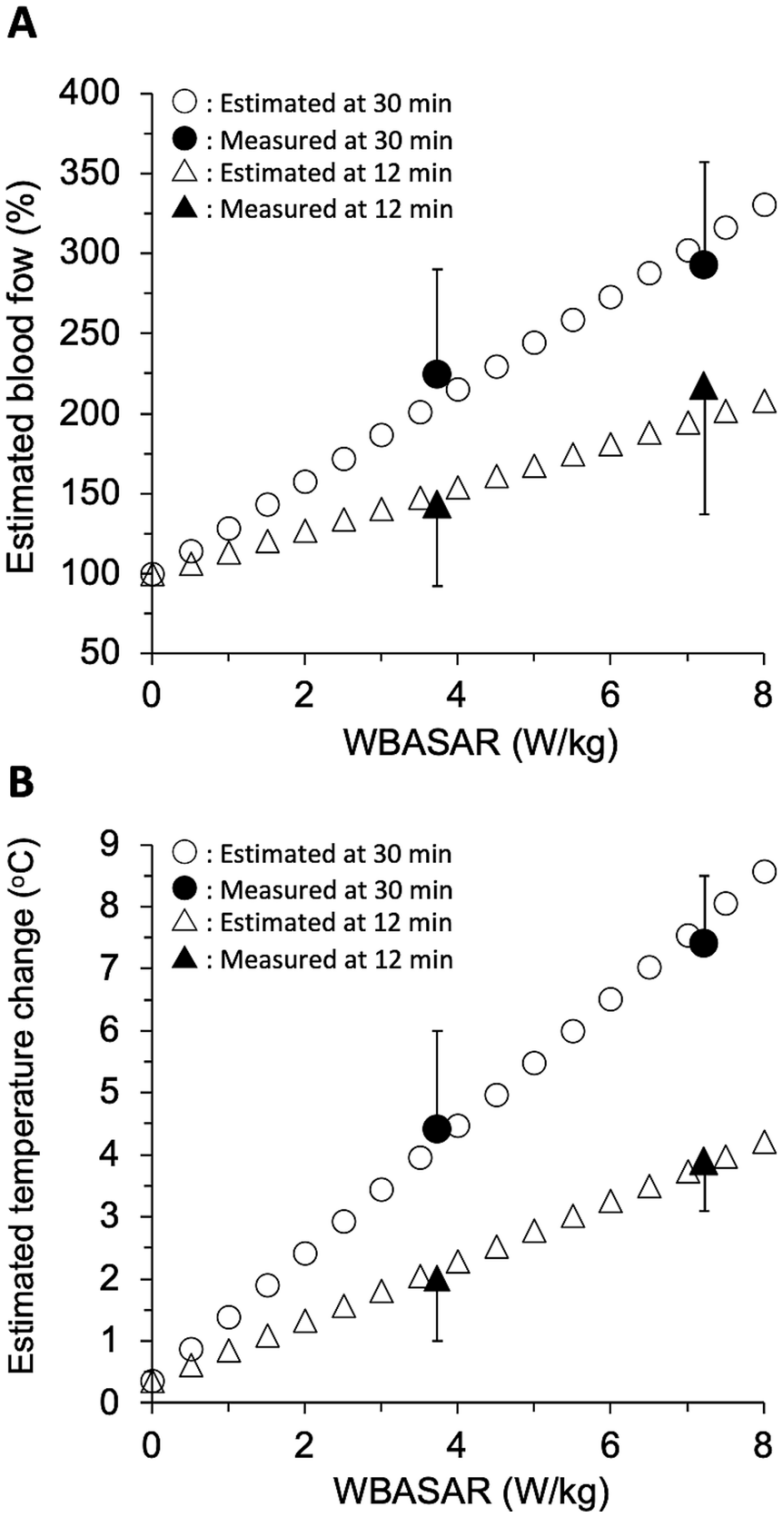
Validation of the thermoregulatory model. Tail skin blood flow **(A)** and temperature change **(B)** were estimated using three regression models with WBASAR ranging from 0 to 8 W/kg at 12 and 30 min of 28 GHz-qMMW exposure. Open plots show each estimate for every 0.5 W/kg up to a maximum of 8 W/kg. Solid plots are values (mean ± SD) obtained from animal studies (Figure 3).

Second, BFt was calculated by substituting the above estimated value of rectal temperature change (Tr) into thermoregulatory model-1. As a result, BFt was estimated to be 100 to 209% and 100 to 331% at 12 and 30 min of exposure, respectively, relative to WBASAR 0–8 W/kg (Figure 7A).

Third, the change in tail skin temperature (Tt) was calculated by substituting BFt estimated in the second step just above into thermoregulatory model-2. As a result, Tt were estimated to be 0.4 to 4.2°C and 0.4 to 8.6°C at 12 and 30 min of exposure, respectively, for WBASAR 0 to 8 W/kg (Figure 7B).

Finally, we confirmed that both the estimated BFt and Tt values were close to the experimental values (solid plots in Figure 7), suggesting that model-1 and model-2 may be reasonable systems for increasing tail skin temperature as thermoregulatory mechanisms.

## 4. Discussion

The present study had three major findings. First, whole-body exposure to 28 GHz-qMMW induces intensity-dependent temperature increases not only in the dorsal skin but also in the rectum and tail skin of rats. Second, these temperature changes accompany blood flow (BF) increases in the tail skin but not in the dorsal skin. Third, thermoregulatory systems can be activated by qMMW exposure due to rectal temperature changes.

### 4.1. Region of heat generation

In this study, we subjected rats to whole-body exposure to 28 GHz-qMMW and measured their physiological parameters using a real-time measurement system for biological responses. We succeeded in simultaneously capturing changes in body temperature and skin blood flow in response to qMMW exposure in awake conditions. The temperature changes in each region appeared with respect to their respective elapsed times. The skin temperature on the dorsal side just below the antenna increased rapidly after exposure, followed by an increase in the temperature in other regions. A similar result was observed in anesthetized rats exposed to 35 or 94 GHz MMW (7, 25). These patterns appear to be consistent with the initial accumulation of energy in the dorsal skin, followed by heat transfer to internal structures. This indicates that the energy of the qMMW is absorbed at the skin surface, which is consistent with the fact that MMW penetrates only a few millimeters through the skin (15, 16).

### 4.2. Empirical estimation of exposure intensities required for expected temperature changes

It is important to estimate the intensity of qMMW exposure to increase tissue/organ temperature, which could cause biological effects. In the ICNIRP guideline for local and whole-body exposures to >6 to 300 GHz, skin and core temperatures to avoid health effects are set at 5°C for 6 min of local exposure and 1°C for 30 min of whole-body exposure, respectively (10). However, little information is available regarding the exposure intensities that induce temperature changes in living animals and humans. This is because the current values of exposure intensities in the guidelines were estimated using simulated human models (12, 13).

The present study related local temperature and exposure intensities in a living rat body and allowed the estimation of the exposure intensity required to reach the expected temperature for exposure, at least under the experimental conditions we used. The estimation revealed that the exposure to 488 W/m^2^ of APD was necessary to increase the dorsal skin temperature by 5°C for 6 min. This intensity was twice or more than that simulated (200 W/m^2^) using the human skin model (16). This suggests that the temperature in the living skin does not increase as much as that in simulated skin, suggesting the occurrence of thermoregulation, such as BF changes. On the other hand, whole body exposure at 4.6 W/kg of WBASAR for 30 min would induce a 1°C increase in rectal temperature in the rat. This intensity was quite similar to that estimated using the human whole-body model (4 W/kg for a 1°C increase in core temperature).

The data presented in Table 3 provide additional information on the following two adverse biological effects. First, in dorsal skin, qMMW exposure at 291 W/m^2^ or more of APD can increase the skin temperature by 5°C for 30 min. At the beginning of exposure, the skin temperature was 34.9 ± 0.9°C. Thus, the final temperature of the dorsal skin was expected to be 40°C. It has been reported that the critical temperature for deep dermal burns was 41.9°C in rat skin (26). Therefore, if the exposure intensity exceeds an APD of 291 W/m^2^, qMMW exposure may induce skin damage. Second, we estimated that qMMW exposure at 38 W/kg of WBASAR increased the rectal temperature by 5°C for 30 min. This suggests that the rectal temperature of the rat reaches 42°C because its initial temperature was 36.7 ± 0.5°C. Several research groups have found a precipitous decrease in arterial blood pressure and subsequent death in rats at approximately 42°C of colonic temperature under MMW exposure (25, 27, 28). Thus, a similar phenomenon may be induced at our estimated exposure intensity of 38 W/kg of WBASAR.

### 4.3. Blood flow proportion in rat body

The rat may have used its tail to dissipate the heat accumulated in its body under 28 GHz-qMMW exposure. It is well known that the rat tails play an important role in releasing heat from its body, acting like a radiator because rats do not use sweat glands (19). In animals, the tail, ears, and limbs are important heat-dissipating organs. When the bodies of rats and guinea pigs were electrically heated and the local temperature was examined, the tail temperature showed a marked increase compared to other organs (29). It has also been confirmed that almost 20% of heat is dissipated from the rat tail (30). In general, the temperature of the rat tail skin is the same as the ambient temperature and blood barely flows from the tip when the tail is amputated. However, when the body is warmed and the temperature of the tail is raised, thick blood vessels become visible under the tail skin and blood starts to flow from the tip if the tail is cut off (29). In other words, when the body is warmed, heat is transported to the tail through the BF.

A similar physiological response was observed in the tail following qMMW exposure. qMMW exposure increased skin temperature and BF in the tail. Moreover, the temperature changes in the tail skin were twice as large as those in the dorsal skin (Figures 3A,C), even though the tail skin received little exposure to qMMW. Interestingly, BF did not change in the dorsal skin even when heated to the maximum intensity of qMMW exposure. These results suggest that the heat generated in the rat dorsal skin is transferred to the tail skin via an intentional change in the proportion of BF to maintain or reduce the core body temperature.

### 4.4. Thermoregulation model and threshold

Whole-body exposure to 28 GHz-qMMW could be a triggered the activation of thermoregulatory systems via an increase in core temperature in rats. To explore the mechanism of thermoregulation observed in rats exposed to qMMW and estimate the threshold of exposure intensity that drives this regulation, we investigated the correlation between the measured parameters. We found that the three linear regression models were suitable for explaining and mimicking these systems. Namely, the models could demonstrate the following steps: (1) heat generation by whole-body exposure to qMMW (which mainly occurs in the skin of the body surface) accumulates in the internal body and increases the core temperature; (2) the change in core temperature reflects the BF increase in the tail skin; and (3) the BF increase in the tail skin is directly reflected in the tail skin temperature increase and promotes heat dissipation from the tail.

Each of these steps was consistent with the findings of previous physiological experiments. The results from Step 1 were supported by Millenbaugh’s findings using ambient temperature increase and MMW exposure (7, 25), although these studies lack an assessment of the intensity dependence of temperature. Step 2, described as model-1, may involve the central nervous system in rats. It is possible that the hypothalamus senses an increase in core temperature (31, 32) and dilates peripheral blood vessels in the tail (33) through the inactivation of the efferent nerve (sympathetic nerve) (34, 35). Step 3, which was explained as model-2, is also shown in the proportional relationship between BF and temperature in the rat tail directly (36) and indirectly (37). Other mechanisms such as evaporative respiratory heat dissipation (38) may also contribute to thermoregulation under qMMW exposure. However, the estimated values of blood flow and temperature changes in the tail skin were similar to those measured in conscious rats. Our findings suggest that these models are dominant in simulating thermoregulation under qMMW exposure.

According to the estimated results using thermoregulatory model-1 (Figure 6A) and model-2 (Figure 6B), whole-body exposure to qMMW may trigger thermoregulatory systems even at less than 4 W/kg of WBASAR. For example, whole-body qMMW exposure at 2 W/kg could drive the thermoregulatory systems, accompanied by increases in tail BF and tail skin temperature by approximately 50 points and 2.5°C, respectively (Figure 7). Many research groups have used 4 W/kg of WBASAR as one of the criteria or thresholds to evaluate the non-thermal/thermal effects of exposure to microwaves in animal experiments (3, 39–46) because this value of 4 W/kg is a biological basis for the basic restriction mentioned above. In addition, some of these studies found biological effects of whole-body exposure below a WBASAR of 4 W/kg (3, 46). However, the biological responses hypothesized here seem to be reversible and physiological to maintain homeostasis (36). Therefore, it is important to confirm whether the effects of low exposure intensity are adverse or within the physiological range.

In the present study, there are a couple of limitations that should be considered when establishing a thermoregulatory model. First, rats were irradiated with qMMW only from the dorsal side. The abdominal skin of the rat, which was placed on the opposite side of the antenna, was not directly exposed. Therefore, the abdominal skin may contribute to the keep/decrease in core temperature. Second, there was large dispersion in the measurement data, particularly for skin blood flow. This was due to the experimental difficulties caused by the use of conscious rats. Nevertheless, the models obtained for physiological parameters could provide useful information for future experiments because of the similarity in values between the experimental measurements and estimations.

## 5. Conclusion

In conclusion, to clarify the effects of 28 GHz-qMMW exposure on the whole body, we focused on changes in local temperatures and skin blood flow. Intensity-dependent temperature increases were observed in the dorsal skin and rectum after qMMW exposure. In addition, the skin blood flow ratio was modified by rectal temperature elevation, resulting in an increase in tail skin temperature. These findings suggested that whole-body MMW exposure drives thermoregulation to transfer the heat generated by exposure to the body surface. Despite the large differences between humans and rats in terms of size and physiology, our findings may be helpful for discussing the basic restriction values in the international exposure guidelines.

## Data availability statement

The original contributions presented in the study are included in the article/supplementary material, further inquiries can be directed to the corresponding author.

## Ethics statement

The animal study was approved by Kurume University School of Medicine (approval numbers: 2020-174 and 2021-150). The study was conducted in accordance with the local legislation and institutional requirements.

## Author contributions

HM: conceptualization. EI and HM: study design. EI, TI, SK, AH, TH, and HM: experimentation. EI, AM, and HM: statistical analysis. EI and HM: writing—original draft preparation. EI, HM, TI, AM, SK, and AH: writing—review and editing. EI and HM: visualization. HM and AH: supervision. All authors contributed to the article and approved the submitted version.

## Funding

This work was financially supported by the Ministry of Internal Affairs and Communications, Japan, under Grant JPMI10001.

## Conflict of interest

The authors declare that the research was conducted in the absence of any commercial or financial relationships that could be construed as a potential conflict of interest.

## Publisher’s note

All claims expressed in this article are solely those of the authors and do not necessarily represent those of their affiliated organizations, or those of the publisher, the editors and the reviewers. Any product that may be evaluated in this article, or claim that may be made by its manufacturer, is not guaranteed or endorsed by the publisher.

## Abbreviations

MMW: Millimeter wave
qMMW: Quasi-millimeter wave
BF: Blood flow
SAR: Specific absorption rate
WBASAR: Whole-body averaged SAR
CW: Continuous wave
IP: Input power
IPD: Incident power density
APD: Absorbed power density
